# Tools for macromolecular model building and refinement into electron cryo-microscopy reconstructions

**DOI:** 10.1107/S1399004714021683

**Published:** 2015-01-01

**Authors:** Alan Brown, Fei Long, Robert A. Nicholls, Jaan Toots, Paul Emsley, Garib Murshudov

**Affiliations:** a MRC Laboratory of Molecular Biology, Francis Crick Avenue, Cambridge CB2 0QH, England

**Keywords:** model building, refinement, electron cryo-microscopy reconstructions, *LIBG*

## Abstract

The recent rapid development of single-particle electron cryo-microscopy (cryo-EM) now allows structures to be solved by this method at resolutions close to 3 Å. Here, a number of tools to facilitate the interpretation of EM reconstructions with stereochemically reasonable all-atom models are described. The *BALBES* database has been repurposed as a tool for identifying protein folds from density maps. Modifications to *Coot*, including new Jiggle Fit and morphing tools and improved handling of nucleic acids, enhance its functionality for interpreting EM maps. *REFMAC* has been modified for optimal fitting of atomic models into EM maps. As external structural information can enhance the reliability of the derived atomic models, stabilize refinement and reduce overfitting, *ProSMART* has been extended to generate interatomic distance restraints from nucleic acid reference structures, and a new tool, *LIBG*, has been developed to generate nucleic acid base-pair and parallel-plane restraints. Furthermore, restraint generation has been integrated with visualization and editing in *Coot*, and these restraints have been applied to both real-space refinement in *Coot* and reciprocal-space refinement in *REFMAC*.

## Abstract 2

A description is given of new tools to facilitate model building and refinement into electron cryo-microscopy reconstructions.

## 1. Introduction   

Single-particle electron cryo-microscopy (cryo-EM) is currently undergoing a technical revolution (Kühlbrandt, 2014 ▶; Smith & Rubinstein, 2014 ▶). This has allowed the structures of macromolecules to be solved at near-atomic resolution (defined in this context as when the density map is sufficiently resolved to build a reasonably reliable full-atom model; Liao *et al.*, 2013 ▶; Allegretti *et al.*, 2014 ▶; Amunts *et al.*, 2014 ▶). The improvement in resolution is predominantly owing to cameras that detect electrons directly and also feature improved quantum efficiencies and readout rates (Faruqi & McMullan, 2011 ▶). The new detectors have ignited developments in EM data processing, including software based on statistical algorithms that classify samples (Scheres, 2012 ▶) and correct for beam-induced sample motion (Li *et al.*, 2013 ▶; Bai *et al.*, 2013 ▶; Scheres, 2014 ▶).

**Figure 1:**
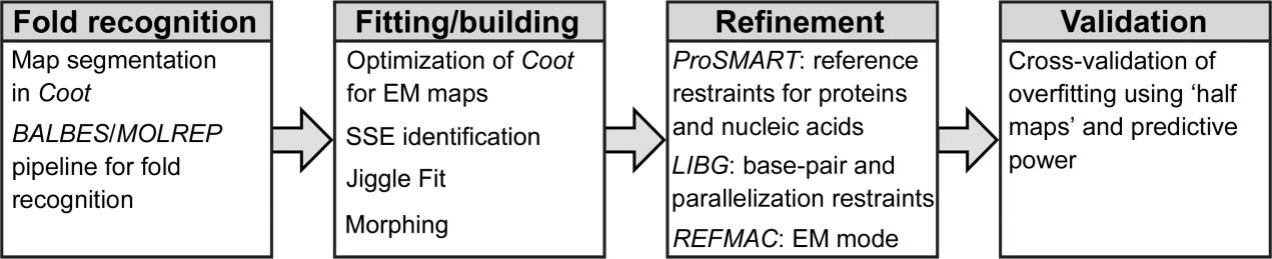
Tools to facilitate the interpretation of EM data with atomic models.

Structural information to near-atomic resolution is necessary to fully understand the detailed molecular mechanisms that underpin biological function. At resolutions of 4.5 Å or better the C^α^ backbone of protein components can be built based on the map alone, and at resolutions better than 4.0 Å amino-acid side chains become apparent. At these resolutions it should be possible to determine all-atom structures to the same degree of accuracy as with crystallographic data sets at similar resolutions. Indeed, since phases and amplitudes are determined equally well in EM, it is expected that models produced through the interpretation of EM density should be more accurate. The fit of the model to density and its consistency with expected chemical and structural knowledge are of equal importance. For this purpose, besides describing tools to facilitate model building, we also describe methods to refine the models using a suite of restraints derived from prior knowledge and to validate the results (Fig. 1 ▶).

## 2. Local resolution heterogeneity   

The overall resolution of a cryo-EM reconstruction is typically measured using the Fourier shell correlation (FSC), which provides a single value for the entire map and depends critically on the threshold criterion used (Rosenthal & Henderson, 2003 ▶; Scheres & Chen, 2012 ▶; Chen *et al.*, 2013 ▶). The ‘gold-standard’ approach to resolution determination requires that during data processing the images are divided into two subsets (preferably at random), each containing one half of the images of the complete set. The FSC between the two maps at a threshold of 0.143 provides the resolution limit of the reconstruction (Rosenthal & Henderson, 2003 ▶). For a discussion of ‘gold-standard’ FSC calculations, please see Scheres & Chen (2012 ▶). However, cryo-EM maps are typically chimeras of regions of highly variable resolution, and a single resolution measurement can be misleading, although useful. Generating a three-dimensional reconstruction is the result of averaging many thousands of individual two-dimensional particle projections; each of these particles is unlikely to be in exactly the same conformation. Samples that have intrinsic flexibility or ligands that are present at less than full occupancy will display lower resolution than rigid regions at full occupancy. Inaccuracies in the alignment of individual particles will also limit resolution. To fully, and correctly, interpret the map it is important to know the resolution to which reliable features extend (Cardone *et al.*, 2013 ▶). In X-ray crystallography, model-building and refinement strategies are selected on the basis of the overall resolution (Nicholls *et al.*, 2012 ▶), but cryo-EM may require ‘multi-resolution modelling’ where separate strategies are employed in different regions of the same reconstruction. These strategies should not overlook data from complementary techniques (for example chemical cross-linking mass spectrometry) at lower resolution (Lasker *et al.*, 2012 ▶).

## 3. Interpreting EM density maps: fold recognition   

One strength of cryo-EM is the ability to determine structures of macromolecular complexes isolated from native sources in low yields. In such cases the individual components within the complex may not be known, as in a recent cryo-EM reconstruction of a ribosome-biogenesis intermediate (Leidig *et al.*, 2014 ▶). Therefore, it is not possible to interpret the maps simply by docking high-resolution structures or comparative/*ab initio* models as this requires the identity of the components to be known; different strategies are required. At resolutions better than 4.0 Å it may be possible to trace the density and build the structure *de novo*; this model could then be used to interrogate the Protein Data Bank (PDB; Berman *et al.*, 2002 ▶) for possible structural matches. If the resolution permits, it may be possible to deduce an amino-acid sequence from the side-chain densities that could be used to search protein-sequence databases. An alternative approach is fold recognition, where the density is searched for features resembling known protein domains and motifs. Two approaches have been described: *FREDS* (Khayat *et al.*, 2010 ▶), which uses a protein-domain parser, *PDP* (Alexandrov & Shindyalov, 2003 ▶), to prepare a library of folds directly from the PDB that are then searched against the density map, and *SPI-EM* (Velázquez-Muriel *et al.*, 2005 ▶), which determines the probability of a CATH-defined superfamily (of which there are currently 2500; Sillitoe *et al.*, 2013 ▶) fitting the density rather than a brute search of a large library of domains.

We have implemented density-based fold recognition using a curated database of protein domains, *BALBES* (Long *et al.*, 2008 ▶), which is not restricted to categorized domains. *BALBES* was originally implemented as an automated molecular-replacement pipeline to use known structures to solve the crystallographic phase problem. While obtaining phases is not a problem in cryo-EM, the database can instead be utilized for screening against unidentified density. While any rigid-body docking program can be used with *BALBES*, we used *MOLREP* (Vagin & Teplyakov, 2010 ▶), which is suitable for accurate high-throughput fitting (Khayat *et al.*, 2010 ▶). Alternative rigid-body docking software has recently been reviewed by Villa & Lasker (2014 ▶).

At its core, the *BALBES* pipeline comprises a non­redundant database of approximately 50 000 protein domains greater than 15 amino acids in length and refined against data extending to resolution limits of better than 3.5 Å. Domains in the *BALBES* database are defined by their three-dimensional compactness and separability from other parts of a macromolecule. All these domains were selected and then trimmed from the existing nonredundant macromolecular subunits in the PDB, among which no two subunits had a sequence identity of greater than 80% and a root-mean-square deviation (r.m.s.d.) between corresponding C^α^ atoms of less than 1 Å. To further reduce the fold redundancy within these domains, we reclassified the domains according to identity of space groups, a similarity of unit-cell parameters of 95% and a sequence identity of 95%. The re-classification was carried out using a modified algorithm of equivalence classes (Press *et al.*, 1992 ▶), full details of which will be published elsewhere. After re-classification, we have approximately 14 000 domains of likely unique folds.

We also provide a new library, *RNA Looplib*, of structural RNA fragments (internal and hairpin loops) based on motif classes taken from the Motif Atlas (Petrov *et al.*, 2013 ▶). Redundancy is reduced by selecting the motif solved at highest resolution for each class. Motifs with fewer than four nucleotides are discarded, leaving approximately 600 unique motifs. The library is updatable for new RNA 3D Motif Atlas releases. *RNA Looplib* is intended to be used in the same way as the *BALBES* database for nucleic acid-containing reconstructions.

**Figure 2:**
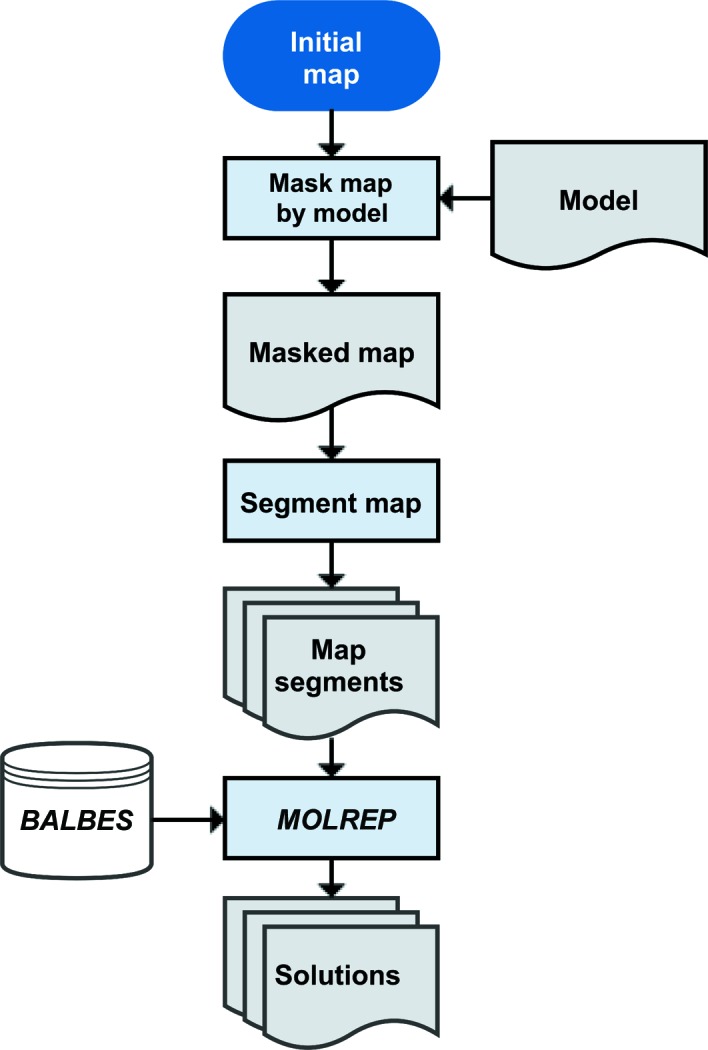
Flowchart of the *BALBES*–*MOLREP* pipeline implemented for fold recognition using map-masking and segmentation tools in *Coot*.

To test the application of the *BALBES*–*MOLREP* pipeline for fold recognition (Fig. 2 ▶), we used the cryo-EM reconstruction (EMD-2566) of the large subunit from the yeast mitochondrial ribosome (hereafter referred to as 54S; Amunts *et al.*, 2014 ▶). As well as regions with homology to bacterial ribosomes, 54S contains a number of mitochondria-specific proteins that after *de novo* building were shown to share structural, but not functional, conservation with proteins of known structure. Using fold recognition, can these structural homologues be identified from the density alone and used to guide model building?

Excluding all density that could be explained by homology to bacterial ribosomes, the supernumerary density was segmented into a library of search maps corresponding to putative individual components. Segmentation can simplify rigid-body docking to a local rather than an exhaustive global search and also assist in *de novo* building by reducing the map size and introducing clearly defined boundaries. Automated, or semi-automated, procedures for map segmentation, for example *Segger* in *Chimera*, remain a considerable challenge for closely packed multi-protein complexes such as ribosomes (Pintilie & Chiu, 2012 ▶). Therefore, we adopted a manual approach of segmenting spherical regions of unidentified density in *Coot*. The rotation centre and radius are user-defined, although we typically found 34 Å to be well suited to the identification of protein domains and 17 Å to be suitable for RNA motifs. To aid visualization of the location of unidentified density in *Coot*, spherical markers can be placed at the rotation centres. Alternatively, *Coot* can mask maps by a set of atom coordinates.

For each domain in the *BALBES* database, *MOLREP* was executed against each map fragment. Default settings were used, specifying that the search solution should be a single molecule and applying a high-resolution limit of 5 Å. The *MOLREP* contrast score was used to identify a correct solution. This represents the difference between the highest and the mean score expressed in terms of standard uncertainty. In molecular replacement of X-ray crystallographic data, a contrast score of higher than 3 is a good indication of a correct solution.

**Figure 3:**
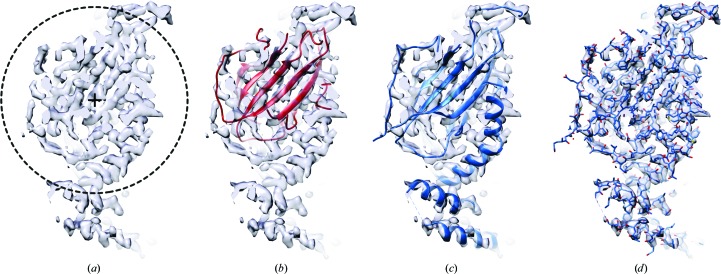
Fold recognition can identify template molecules for model building. (*a*) Density map corresponding to the final model of the mitoribosomal protein mL38 with the segmented search map indicated. (*b*) Top solution from the *BALBES*–*MOLREP* pipeline. (*c*, *d*) Final refined model of mL38 in (*c*) cartoon and (*d*) full-atom representation.

Taking a single map fragment as an example, the best solution was a phosphatidylethanolamine-binding protein (PEBP) from mouse (PDB entry 1kn3; Simister *et al.*, 2002 ▶), with a contrast score of 6.9. As only one mitoribosomal protein (mL38) was predicted to contain a PEBP superfamily domain, this section of the map could be assigned and the solution used as a template to build the protein *de novo* (Fig. 3 ▶). Alternatively, the solution could be used as a template for automated rebuilding using programs such as *Rosetta* (DiMaio *et al.*, 2009 ▶). After rebuilding, the structure of mL38 (PDB entry 3j6b, chain 1; Amunts *et al.*, 2014 ▶) was used to identify structural homologues in the PDB (Krissinel & Henrick, 2004 ▶), with the best match sharing the same fold as 1kn3 (PDB entry 1wpx; Mima *et al.*, 2005 ▶) but resolved at a lower resolution. This confirms that the *BALBES*–*MOLREP* pipeline identified the best possible solution from over 14 000 domains. That the search density did not correspond exactly with the density belonging to mL38 demonstrates that the technique is not reliant on stringent or accurate segmentation. However, integrating automated segmentation with the *BALBES*–*MOLREP* pipeline should facilitate the rapid population of density as an initial step to fully automated map interpretation. The pipeline is equally suited to searching for protein folds in crystallographic maps where only a partial solution is known.

## 4. Model building   

*Coot* is an interactive three-dimensional modelling program designed for the building and validation of macromolecular structures with a particular emphasis on processes that require manual intervention (Emsley & Cowtan, 2004 ▶). In EM, *Coot* has been utilized as a tool for improving the initial fit and also for *de novo* model building; however, the program had not been optimized for this. To improve the functionality of *Coot* for EM, we have implemented a number of new tools (detailed below) that are also applicable to X-ray crystallography.

### 4.1. Jiggle Fit   

Jiggle Fit is implemented to be used downstream of either rigid-body docking or manual placement of domains and secondary-structure elements (SSEs) to improve the fit to the density. Prior to this work, *Coot* had an extant simple ‘Jiggle Fit’ system that was designed to optimize the orientation of small ligands (Debreczeni & Emsley, 2012 ▶). The atom selection was restricted to a single residue, no map masking was performed and there was no consideration of the neighbouring atoms that might affect the pose. The original system applied a random set of rotations and translations to generate hypotheses, each of which was scored using a *Z*-weighted sum of the map density at the atom positions. The rotations were selected from a uniform distribution on (0, 2π) for each of the three independent rotation axes, and translations along each of the axes were selected from a uniform distribution on (0, *s*), where *s* is a user-definable distance. The model with the highest scoring fit to the density then underwent real-space refinement before the coordinates were updated. This system was extended to make it suitable for optimizing the fit to density for macromolecules as follows.

(i) The atom selection was generalized to allow arbitrary selection (for example atoms of a single chain).
(ii) The addition of a scaling factor for the transformations: as the function progresses the rotation range available is reduced by a factor of *c*/*N* (where *c* is the current cycle number and *N* is the number of rotation–translation trials). The translations are similarly downscaled. This increases the probability of generating hypotheses that are ‘close’ to the original.
(iii) Each solution is scored by its fit to the density (by the *Z*-weighted sum of density at atom centres). Instead of automatically selecting the top-fitting transformation, the top solutions (by default, 20) are individually fitted into the map by rigid-body refinement. The solutions are re-scored by their fit to the density, and if the best-fitting molecule is a better fit than the original then the coordinates are updated.

To test the dependence of Jiggle Fit on map resolution, we created reconstructions of the 54S subunit at multiple resolutions ranging from 3.4 to 6.8 Å (Table 1 ▶). Rather than low-pass filtering the maps to lower resolution, we generated maps with subsets of particles using *RELION* (Scheres, 2012 ▶) to more closely replicate real data sets. The coordinates for a reference molecule (bL9) were agitated as a rigid body by both a random set of unlimited rotations around each axis and a random set of translations that were limited to a defined distance from the final coordinates (0–5 Å). Jiggle Fit was then performed at each resolution for all starting models and the output was assessed by superposition with the reference model. The trials were conducted using complete 54S maps, rather than with segmented maps, to replicate instances in which the boundaries of the protein are not fully known.

**Figure 4:**
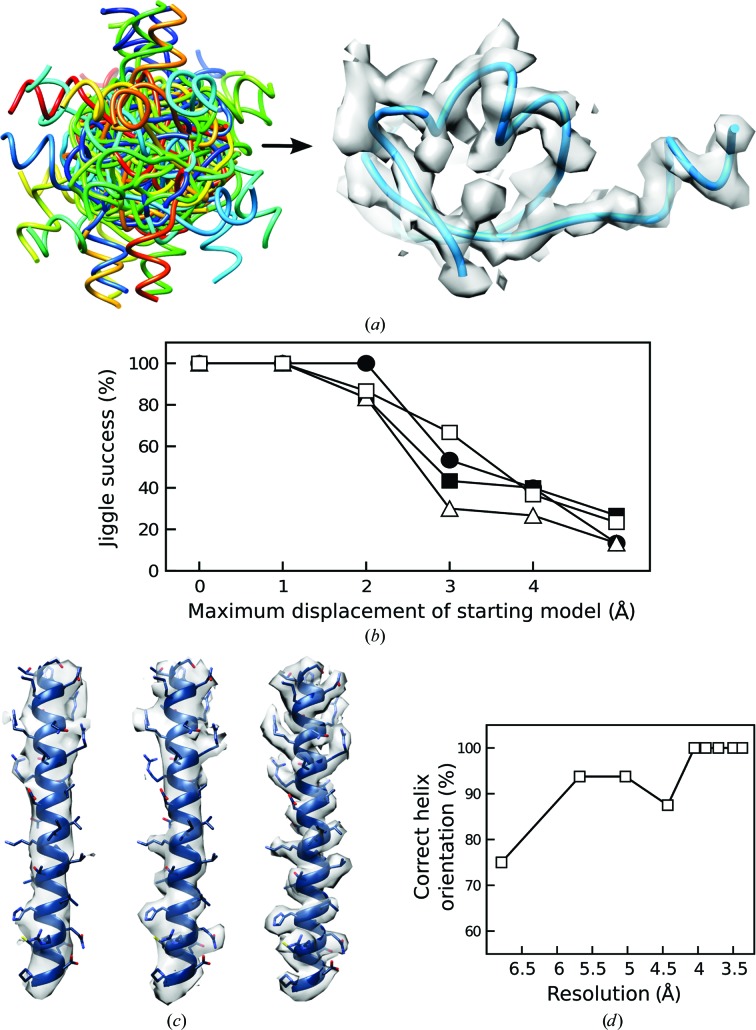
(*a*, *b*) Jiggle Fit improves local fit to density. (*a*) Randomly rotated and displaced models (by up to 1 Å, left) can be jiggled into their corresponding densities in a manner not dependent on resolution (right). (*b*) The dependence of Jiggle Fit on resolution and displacement from the correct solution. For clarity, four resolutions are shown: 3.37 Å (unfilled squares), 4.05 Å (triangles), 5.03 Å (circles) and 6.79 Å (filled squares). (*c*, *d*) Jiggle Fit coupled to SSE identification. (*c*) Examples of density for an α-helix at (from left to right) 6.8, 5.0 and 3.2 Å resolution, showing loss of pitch and side-chain densities at lower resolution. (*d*) Resolution dependence of Jiggle Fit in determining helix orientation.

From the results, translation had a greater effect on the rate of success than rotation (Figs. 4 ▶ *a* and 4 ▶ *b*). Jiggle Fit identified the correct solution for each attempt where the coordinates were randomly rotated, or randomly rotated and displaced by up to 1 Å in any direction. As the position of the starting model diverges further from the final location, Jiggle Fit is less able to determine the correct solution. Even at a resolution close to 7 Å and displaced up to 5 Å from the final position, the correct solution is successfully attained in 20% of cases.

### 4.2. Morphing   

Often, the initial model used to interpret the density map is similar to the structure to be solved. However, differences, perhaps as the result of conformational changes, the absence of crystal contacts or inaccurately modelled regions, can leave sections of the model outside the density. Additionally, rigid-body docking of multiple components can result in unphysical bonds and steric clashes at the boundaries of domains. To overcome some of these limitations, fitting methods have been described that take into account the dynamic properties of macromolecules. These include normal modes, as implemented in *iMODFIT* (Lopéz-Blanco & Chacón, 2013 ▶), deformable elastic networks, as in *DireX* (Wang & Schröder, 2012 ▶), and molecular-dynamics flexible fitting (*MDFF*; Trabuco *et al.*, 2008 ▶).

Model morphing in *Coot* is designed to take advantage of the local similarity of the template and target structures. EM maps are sufficiently noisy and low resolution that a rigid-body fit of individual residues would result in a model with severe geometric problems. The model-morphing tool was designed to make local shifts that reduce geometric distortions. The method takes each residue in turn and constructs a (by default) five-residue fragment based around this central residue (using two residues upstream and downstream of the central residue). Each five-residue fragment is fitted to density by a rigid-body fit, which provides a rotation–translation operator for each residue. Each residue has a local environment (*i.e.* residues which have atoms that are within a user-specified distance, typically 10 Å, of the atoms of the central residue). The rotation–translation operators of the residues of the environment are sorted by how much they move their atoms and robustly averaged, with the top and bottom 25% discarded to provide a rotation–translation operator for the central residue. This process is repeated for each residue in the chain and can be carried out recursively. Indeed, serial application of morphing is often required for convergence. The larger the averaging radius, the smaller the local shifts that are applied, which increases the number of times that this morphing procedure has to be executed to reach convergence.

**Figure 5:**
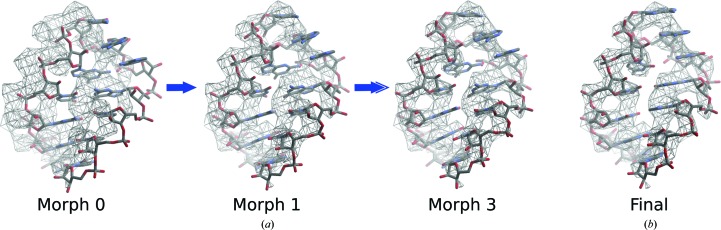
Example of model morphing. (*a*) Section of RNA taken from the complete rigid-body docking of bacterial rRNA into the mitochondrial ribosome map (morph 0) and morphed in *Coot* for three iterations. (*b*) The final refined structure of mitochondrial rRNA.

**Figure 6:**
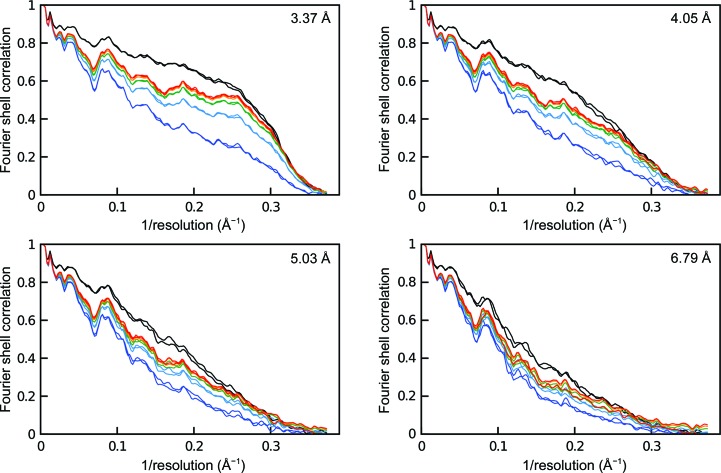
FSC curves following the progress of morphing at 3.37, 4.05, 5.03 and 6.79 Å resolution. Black lines represent the fit of mitochondrial rRNA to both mitochondrial half maps at the given resolution. Dark blue lines represent the initial fit of bacterial rRNA to both mitochondrial half maps. The bacterial rRNA was morphed four times: iterations 1 (light blue), 2 (green), 3 (orange) and 4 (red). Excluding the fourth iteration at 6.79 Å resolution, the FSC curves for both half maps overlap, demonstrating that morphing does not result in overfitting.

To illustrate morphing, the structure of bacterial 23S rRNA (PDB entry 3v2d, chain *A*) was fitted by global rigid-body docking to the density of half maps from 54S reconstructions at resolutions ranging from 3.4 to 6.8 Å (Table 1 ▶). The core regions of rRNA from bacterial and mitochondrial ribosomes are structurally conserved but divergent in sequence, and display local conformational changes at the periphery. There are several regions where the bacterial rRNA model and mitochondrial rRNA density do not correspond, but it is clear that with a relatively small local rotation–translation the residues of the model could be made to fit the map. The bacterial structure was morphed, using a local environment set at 7 Å, for four iterations (Fig. 5 ▶). The progress of morphing was followed by calculating FSC curves for the starting bacterial model, the morphed model and the final fully refined mitochondrial rRNA against the half map used for morphing (FSC_work_). To confirm that morphing was not resulting in overfitting (see below), the FSC was also calculated against the half map that had not been used for morphing (FSC_test_; Fig. 6 ▶).

A similar approach to morphing has been reported (Terwilliger *et al.*, 2012 ▶) for improving crystallographic models, particularly for molecular-replacement solutions that are not close enough to the target structure for automated building, using electron-density maps. Morphing in *Coot* can be used in a similar way.

### 4.3. Identification of secondary-structure elements (SSEs)   

At subnanometre resolutions, SSEs are discernible in density maps: α-helices appear as long cylinders and β-sheets as continuous and somewhat flat expanses of density. As SSEs can reliably be identified from protein amino-acid sequences, locating these in the density map is critical for initiating *de novo* model building. SSE localization has been implemented in both *Gorgon* (Baker *et al.*, 2012 ▶) and *Chimera* (Pettersen *et al.*, 2004 ▶) through a graphical version of *SSEHunter* (Baker *et al.*, 2007 ▶). A similar function, the ‘Find Secondary Structure’ tool in *Coot*, performs a six-dimensional rotation and translation search to find the likely positions of both α-helices and β-strands within the density map (Emsley *et al.*, 2010 ▶).

However, this tool had been tuned to fit to electron-density maps from X-ray crystallography, where there is little variation in the *Z*-score (the number of standard deviations) of the electron density of secondary-structure main-chain atoms. The density maps obtained in cryo-EM reconstructions can have substantially larger *Z*-values owing to the typically larger box size, much of which is filled with zero, or near-zero, density values (a result of putting the EM reconstruction density in an empty box and normalizing). Thus, the calculation of map statistics from EM maps has been changed; instead of simply summing the density values and their squares to generate the mean and variance, the values are now added into finely sampled bins. The peak of this histogram is determined and the corresponding density points are discarded from the calculation of the mean and variance. This results in an estimation of the mean and variance of the map that is more consistent with those from X-ray data and allows the fitting of SSEs, without user intervention, in maps from both X-ray crystallography and cryo-EM.

For nucleic acid macromolecules, *Coot* can generate idealized atomic models with canonical Watson–Crick base pairing of single-stranded or double-stranded A-form or B-form DNA or RNA given a nucleotide sequence. Alternatively, RNA motifs can be obtained from *RNA Looplib* or modelled using *Assemble*2 (Jossinet *et al.*, 2010 ▶) and imported into *Coot*. These can all act as starting points for *de novo* building.

After the localization of SSEs and/or idealized nucleic acid helices, Jiggle Fit can be used to improve the fit to density and to correctly orientate α-helices. To demonstrate this, we placed polyalanine helices in both orientations in density corresponding to the mitoribosomal protein bL27 (PDB entry 3j6b, chain *R*). Each helix was subjected to Jiggle Fit and scored for correct orientation against the final structure for a range of resolutions. The results (Fig. 4 ▶) show that at up to 4 Å resolution helix identification followed by Jiggle Fit invariably finds the correct orientation; even at close to 7 Å resolution, where helices predominantly appear as featureless tubes (Fig. 4 ▶), the correct orientation is identified 75% of the time.

### 4.4. *De novo* building   

*Coot* offers many tools for *de novo* model building. C^α^ baton mode allows the path of a protein to be traced by placing correctly spaced C atoms that can then be converted into a main chain and the sequence assigned (Emsley *et al.*, 2010 ▶). Alternatively, residues can be added to the N-termini and C-termini of chains one residue at a time. For building nucleic acids, *RCrane* (Keating & Pyle, 2012 ▶) allows users to trace the backbone by placing phosphates into density and then automatically constructs all-atom models of the nucleotides. Once an initial model has been built, *Coot* has a suite of tools for moving atoms to optimize the fit and stereochemistry, alongside methods of validation (Emsley & Cowtan, 2004 ▶; Emsley *et al.*, 2010 ▶).

## 5. Refinement   

Model refinement is performed to maximize the agreement between the model and experimentally observed data and to minimize stereochemical violations. Refinement in this sense should not be confused with three-dimensional map refinement, but refers to the optimal fit of an atomic model into the density map. In model refinement, atomic coordinates, *B* factors and occupancies are typically adjusted, amongst other parameters. In X-ray crystallography, refinement is performed iteratively alongside automated and manual model building to improve the model and also to calculate electron-density maps, which are then subsequently used to aid further model building. *REFMAC* (Murshudov *et al.*, 2011 ▶) utilizes maximum likelihood to minimize a two-component target function, with one component utilizing geometry (or prior knowledge) and the other utilizing the fit to the experimental data. The relative contribution of these two components can be adjusted by specifying a weight.

*XPLOR*-*NIH* (Maki-Yonekura *et al.*, 2010 ▶), *CNS* (Cheng *et al.*, 2011 ▶) and *phenix.refine* (Baker *et al.*, 2013 ▶) have previously been used for refinement of models into cryo-EM data by adopting a pseudo-crystallographic approach. However, many structures deposited alongside high-resolution (4 Å or better) cryo-EM reconstructions have not been refined and consequently have worse stereochemistry than crystal structures solved at similar resolutions. To facilitate the refinement of structures solved by cryo-EM, we have implemented an EM mode in *REFMAC* that allows users to access tools originally designed for refinement of crystallographic data, as well as tools specifically designed to address the unique challenges posed by EM data.

### 5.1. Similarity of real-space and reciprocal-space refinements   

There is some debate in the structural biology community as to whether real-space or reciprocal-space (Fourier space) refinement should be used for optimizing the fit of atomic models into EM maps. Both have their advantages, and in essence refinements in real and reciprocal space are similar (Appendix *A* [App appa]). The advantages of using reciprocal-space refinement are as follows.

(i) All parameters of the model can be refined simultaneously using all data.
(ii) Resolution-dependent weights can be designed, in particular using maximum-likelihood refinement based on Luzzati’s distribution (Luzzati, 1952 ▶). If the variances of ‘observed’ reciprocal-space structure factors are known then they can be used to adjust weights by inflating the corresponding overall variances.
(iii) Existing crystallographic software that can incorporate phase information can be utilized. This means that chemical, structural and ‘jelly-body’-type as well as local molecular symmetry (known in crystallography as noncrystallographic symmetry; NCS) restraints immediately become available for the refinement of models against EM data.
(iv) Overall quality indicators such as FSC_average_ (see Appendix *B* [App appb]) are available as a by-product of refinement.

However, real-space refinement also offers many attractive features.

(i) It is local, and local adjustments such as rotamer search and secondary-structure search can be performed very quickly.
(ii) If the local variances of maps are known then they can be used to design weights for the least-squares minimization function.
(iii) If local variances in real space are known then they can be used to adjust weights for reference-structure restraints.
(iv) It can be, and is, used as a part of human-aided optimal fit into the density.

**Figure 7:**
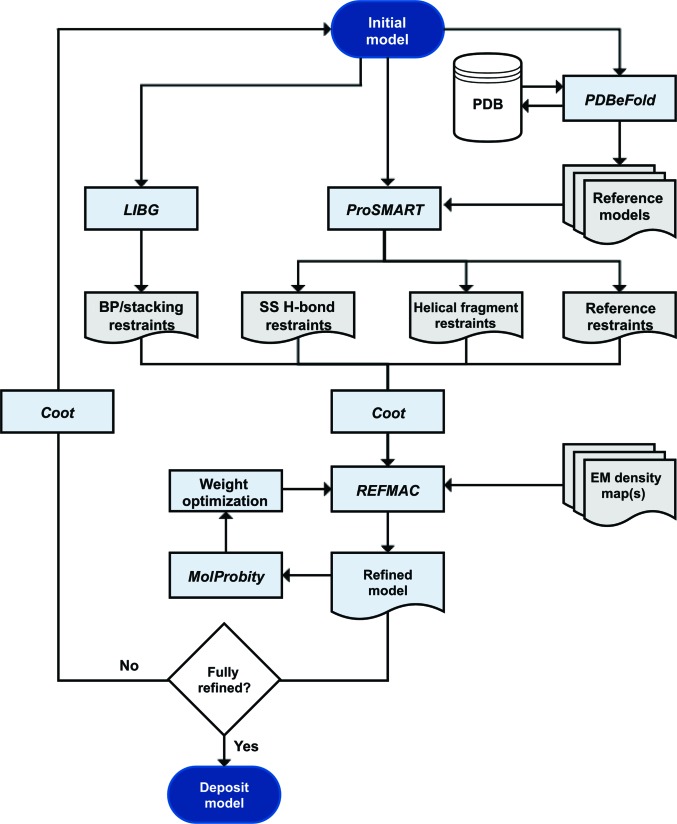
Flowchart showing the overall scheme for restrained refinement of models against EM data. *ProSMART* generates three classes of restraint: (i) reference restraints, (ii) helical fragment restraints and (iii) secondary-structure hydrogen-bond restraints (which include helix, sheet and loop restraints). Alongside reciprocal-space refinement in *REFMAC*, real-space refinement tools in *Coot* can be used to optimize the fit to density.

It has been shown that real-space refinement as a supplement to reciprocal-space methods improves protein models more than the exclusive use of reciprocal space (Chapman & Blanc, 1997 ▶). Therefore, we advocate a strategy that utilizes both real-space refinement tools in *Coot* and reciprocal-space refinement with *REFMAC* (Fig. 7 ▶).

### 5.2. Electron scattering   

Although the density distributions obtained from X-ray crystallography (electron density) and EM (Coulomb potential) both originate from scattering events on the atoms within macromolecules, they are not equivalent. Electrons are scattered by the charge on the nucleus screened by the electron shell of atoms and, unlike the scattering of X-rays, their scattering is affected by local electric charges and ionization states. To take this into consideration, *REFMAC* was modified so that in EM mode it switches to a five-Gaussian approximation for electron scattering factors taken from Cowley *et al.* (2006 ▶).

### 5.3. Refinement against averaged, composite and segmented maps   

Sample heterogeneity can result in multiple maps being calculated from a single data set, with each map displaying discrepancies in both resolution and occupancy (Fernández *et al.*, 2014 ▶; Unverdorben *et al.*, 2014 ▶). The resolution of defined regions within the maps (for example a bound factor or an individual ribosomal subunit) can be improved by focusing particle classification/alignment on this particular region through the application of soft masks during EM data processing (Amunts *et al.*, 2014 ▶; Fernández *et al.*, 2014 ▶). This further expands the collection of maps that can be utilized for model building, refinement and biological interpretation. Multiple maps can be used in refinement to improve the quality of the data to which the model is fitted. Therefore, *REFMAC* has been adapted to handle, and refine against, multiple input maps. Averaging maps will improve the signal-to-noise ratio by increasing the strength of the signal relative to noise. However, in the case of maps generated through focused alignments, averaging may not be desirable as this would negate the advantage introduced by masking. Therefore, *REFMAC* can generate and refine against composite maps formed by combining maps, with averaging only at the interfaces between the maps.

For refinement, *REFMAC* can calculate structure factors for only the section of the map explained by the input model. These are complex structure factors and not just amplitudes, so phase information is not discarded. It is against these structure factors that the model is refined rather than the complete map. This strategy can be used to refine individual components within a larger reconstruction or repeat units of symmetric macromolecules, and requires the model to be placed in a unit cell with the same dimensions as the box size used for the EM reconstruction.

### 5.4. External restraints   

Including chemical and structural information as restraints in refinement reduces the effective number of parameters, thus increasing the effective residual degrees of freedom. Restraints can increase the consistency of the derived atomic models with the available prior knowledge, help to preserve the correct geometry in cases where local structures would otherwise be distorted during refinement, stabilize refinement and reduce overfitting. We have previously demonstrated the value of distance restraints generated from homologous reference structures and structural fragments in improving the quality of protein structures from crystallographic data (Nicholls *et al.*, 2012 ▶, 2013 ▶). It has recently become apparent that their application to EM data is just as valuable (Amunts *et al.*, 2014 ▶). To improve the geometry of nucleic acids during refinement, we have modified *ProSMART* to generate nucleic acid reference restraints, and provide a new tool *LIBG* to generate base-pair and parallel-plane restraints.

### 5.5. *ProSMART*   

Restraints generated using external structural information should help the macromolecule under refinement to adopt a conformation that is more consistent with previous observations. If the reference and target models share a high degree of structural similarity, then we might expect their local inter­atomic distances to be approximately equal. Such information is exploited by *ProSMART*, which generates local interatomic distance restraints that can then be used to aid the refinement of the lower resolution structure in reciprocal space with *REFMAC* or in real space with *Coot*. *ProSMART* only generates restraints with objective values less than a given threshold (typically 4.2 Å) to maintain a degree of global conformational independence between the target and reference structures. Indeed, external restraints are designed to be longer range than chemical bond and angle restraints, while being sufficiently short to be resistant to differences in global conformation. This allows external restraints to be used even when the target and reference structures are, for example, in different bound states, display large-scale domain movements or when crystal contact-induced conformations have resulted in differences between the X-ray and EM structures.

Structurally similar models that can act as reference structures can be identified from the PDB using services such as *PDBeFold* (Krissinel & Henrick, 2004 ▶) or *DALI* (Holm & Rosenström, 2010 ▶). The modifications to *ProSMART* allow reference structures to be either protein and/or nucleic acid macromolecules. As the usefulness of external restraints is limited by the quality of the prior information, reference-model reliability should be considered. The reference structure should be solved experimentally at a higher resolution than the current model and the potential for reference-model errors should not be overlooked. Alongside manual checking of the fit of the model to the density, it may be sensible to attempt re-refinement, and even manual rebuilding, of any reference structure before restraint generation. This might be performed manually or automatically, for example with *PDB_REDO* (Joosten *et al.*, 2009 ▶). Such approaches may reduce error propagation from reference to target models.

*ProSMART* is also able to generate restraints based on generic hydrogen-bond patterns and idealized structural fragments (Fig. 7 ▶). These can help to stabilize protein secondary structure and might be applied when a suitable reference structure is not available, or when the reference is itself not sufficiently well resolved. For example, an ideal α-helix may be used to generate restraints that will keep helical structures intact. Such helical restraints are different to generic hydrogen-bond helical restraints, since they include restraints between all sufficiently close backbone atoms. Also, the fragment-based helical restraints do not require strict compliance with ideal secondary-structure conformation in order to be detected. This is particularly relevant at lower resolutions, where secondary structure may not be sufficiently well formed to be detected from predicted hydrogen-bonding patterns.

The exact usage of external restraints tends to vary between cases and at different stages of the structure-determination process. For example, restraints can be used to temporarily force the maintenance of sensible conformations during the earlier stages of structure determination, and then subsequently to stabilize refinement in later stages. However, it should be acknowledged that such an approach can introduce bias, resulting in the model adopting a conformation that is less consistent with the observed data. However, the use of external restraints can result in a model adopting a conformation very similar to a high-resolution homologue, ideally resulting in an improved model. We suggest that external restraints should only be used if the benefits of any improvements in reliability are deemed to outweigh the negative effects.

### 5.6. *LIBG*   

*LIBG* produces restraints to maintain nucleic acid geometry using information extracted directly from a model, similar to that described for *CNS* and *phenix.refine* (Laurberg *et al.*, 2008 ▶). These restraints are applicable to all DNA/RNA molecules and can be applied in conjunction with reference restraints. Putative base pairs are identified by inspecting the local neighbourhood around the N and O atoms of a base for hydrogen-bond candidates in an adjacent base. A base pair is selected if the combination of hydrogen-bonding patterns between two bases satisfies the preset patterns of hydrogen bonding between DNA/RNA base pairs and the values of the hydrogen-bonding lengths, torsion angles and features of chirality are within the allowed deviation ranges from the corresponding reference values, which are estimated statistically from the database of high-resolution X-ray and neutron crystal structures (Clowney *et al.*, 1996 ▶; Xin & Olson, 2009 ▶). Users can adjust these criteria by changing the allowed deviations.

**Figure 8:**
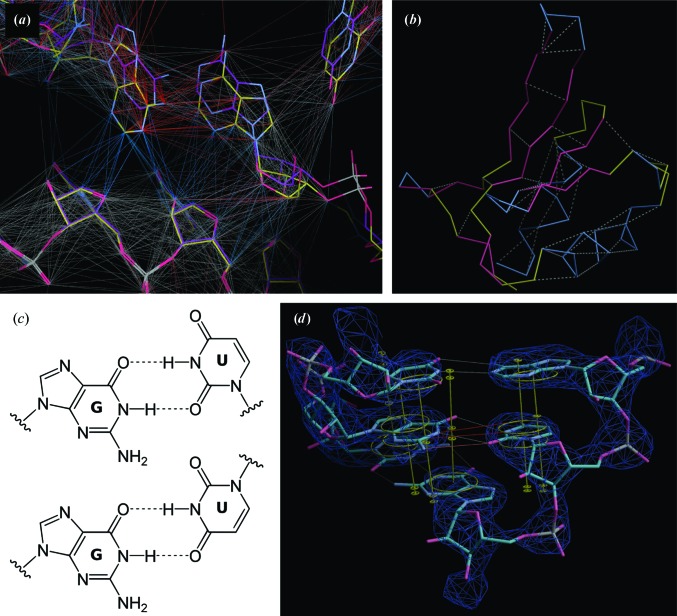
Restraint visualization in *Coot*. (*a*) Restraints were generated using *ProSMART* for an initial model of the mitoribosome (yellow) using the bacterial ribosome (purple) as a reference and were visualized in *Coot*. There are conformational differences between the two rRNA chains despite the sequence identity in the displayed region. Consequently, the local interatomic distances are conserved along the chain (grey) but are shorter across the chain (blue). Interatomic vectors coloured red indicate that the distances in the target structure are longer than in the reference structure. (*b*) Visualization of *ProSMART* hydrogen-bond restraints in *Coot*. (*c*) G:U base pair shown in (top) wobble and (bottom) reverse wobble configuration. (*d*) A G:U base pair with both pairs of *LIBG* restraints displayed in *Coot*. Only the distance restraints that best describe the orientation of the bases (grey, G:U wobble) are used as targets during refinement. Restraints for the reverse wobble configuration are shown in red. Parallel-plane restraints are shown in yellow.

Currently, *LIBG* generates restraints for canonical Watson–Crick and noncanonical G:U base pairs. Since noncanonical base pairing allows multiple pairing of bases (for example, wobble and reverse wobble G:U pairs), *REFMAC* was adjusted to refine against multiple distance and torsion-angle targets (Fig. 8 ▶). During refinement, in every cycle, the best agreeing target is selected as the ‘ideal’ parameter.

*LIBG* also generates restraints to preserve stacking interactions between nucleic acid bases and planar side chains of protein amino acids (parallel-plane restraints). The definition of a plane by a set of atoms is given in Appendix *C* [App appc]. The atom sets appearing in each of all possible planes in DNA/RNA bases and protein residues are also pre-defined (Vagin *et al.*, 2004 ▶). The possible pair of stacking planes is determined by calculating the angle between the normals of two atom planes in different DNA/RNA bases or protein amino acids, angles between the normal of one plane and the vector linking the two ‘gravity’ centres of planar atoms, and the distance between those two ‘gravity’ centres. If the calculated values are within pre-defined ranges, which can be varied by the user, then the two planes are selected as candidates for stacking.

### 5.7. Coupling restraint weight with local resolution   

Unlike the global refinement weight applied during refinement with *REFMAC*, external restraints operate locally. This is of particular use in refinement against EM data, where the most appropriate refinement strategy should be selected based on local resolution. For regions at lower resolution it may be necessary to increase the contribution (weight) of the external restraints in order to restrict overfitting or distorting geometry, whereas for regions of higher resolution the contribution of the external restraints can be reduced to limit model bias. Resolution can be quantified on a local basis using *ResMap* (Kucukelbir *et al.*, 2013 ▶) or by calculating the ‘gold-standard’ FSC while applying a soft mask over the required region. For this purpose, we provide a script that uses *RELION* (Scheres, 2012 ▶) to calculate the local resolution for every chain in a given PDB entry. This information can then be used to select appropriate external restraint weights.

### 5.8. Visualizing external restraints with *Coot*   

Before and after refinement, it is important to manually inspect the model alongside the density map to ensure the local suitability of the use of external restraints. *ProSMART* comparative structural analysis (Nicholls *et al.*, 2014 ▶) can be used to quickly and easily visualize the extent of local conformational changes that occur during refinement. This can provide information regarding stability during refinement, the effect of different refinement protocols and the degree of influence of any external restraints used. If there are any serious artefacts that arise owing to bias towards reference structures, it may be appropriate to re-attempt refinement excluding particular restraints. *Coot* can help to facilitate such manual intervention in the external restraint-generation and restraint-application procedure. Both *ProSMART* and *LIBG* have been integrated with *Coot*. *ProSMART* can be executed directly from within *Coot*, requiring both the target and reference structures to be specified. Any set of externally generated restraints can be visualized and applied in *Coot*, with options for manual editing (Fig. 8 ▶). Restraints corresponding to interatomic distances that are reasonably similar in both models will aid refinement by acting as regularisers, while those exhibiting large differences will have little effect on refinement owing to being weighted down by the use of the Geman–McClure robust estimation function (Geman & McClure, 1987 ▶) in *REFMAC*.

### 5.9. Refinement of symmetric particles   

For symmetric macromolecules, the signal-to-noise ratio can be greatly improved by averaging symmetry-related projections. This typically results in higher resolution reconstructions than can be achieved for asymmetric molecules. By applying symmetry during particle averaging, each asymmetric unit is considered to be identical. It is therefore necessary to refine only a single asymmetric unit against a masked (segmented) map and then apply symmetry operators to generate the complete structure. However, refinement must take symmetry into consideration in order to optimize the contacts at the interface between asymmetric units. Symmetry operators can be given either as a set of operators that generates the whole symmetry group of a molecule or by specifying polar angles, Euler angles or matrix vectors. Once all local symmetry operators are known they are used to generate the symmetry-related atoms that can make nonbonded interactions with the refined molecule, and their contributions to the refinement procedure are included. If the whole map is used for refinement then symmetry-related atoms are used both for map calculation and for the contribution of the fit to the experimental map.

### 5.10. Monitoring fit to density during refinement   

In X-ray crystallography, the *R* factor is a measure of the agreement between the structure amplitudes calculated from a model and those from the data. It is an important global measure characterizing the quality of an X-ray structure for a given set of experimental data. Weighted *R* factors (1) are often used to control behaviour during refinement. However, when weights in refinement change these indicators may not comparable, as demonstrated in Appendix *B* [App appb]. For example, using map sharpening during refinement is equivalent to multiplication of the structure factor **F**_**h**_ by exp(−*Bs* ^2^/4). Therefore, care should be taken when using overall *R* factors, or overall weighted FSCs, as a global measure of fit to density. In order to maintain consistency with crystallographic refinement, *R* factors are calculated using amplitudes of structure factors only, whereas FSC is calculated using complex Fourier coefficients; thus, FSC carries more information about the fit of atomic model parameters into the EM map.
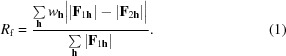
To avoid this dependence on weight, we prefer to use FSC_average_,
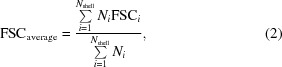
where *N*_shell_ is the number of resolution shells used to calculate FSC, FSC_*i*_ is for the *i*th shell and *N_i_* is the number of structure factors in the *i*th shell. FSC_average_ is therefore independent of weight if the resolution shells are sufficiently thin that the weights on all structure factors within each shell are approximately equal. Average *R* factors would also be less dependent on weight than overall *R* factors; however, they would also be, in general, larger than overall *R* factors. Therefore, to avoid improper usage and comparison between the two values, it would be desirable for FSC_average_ to be adopted as the preferred metric for monitoring the progress of refinement and comparison between structures solved by EM. It should be noted that FSC_average_ is not meant as a replacement for a plot of the FSC between map and model *versus* resolution.

### 5.11. General application of *REFMAC* to EM structures   

**Figure 9:**
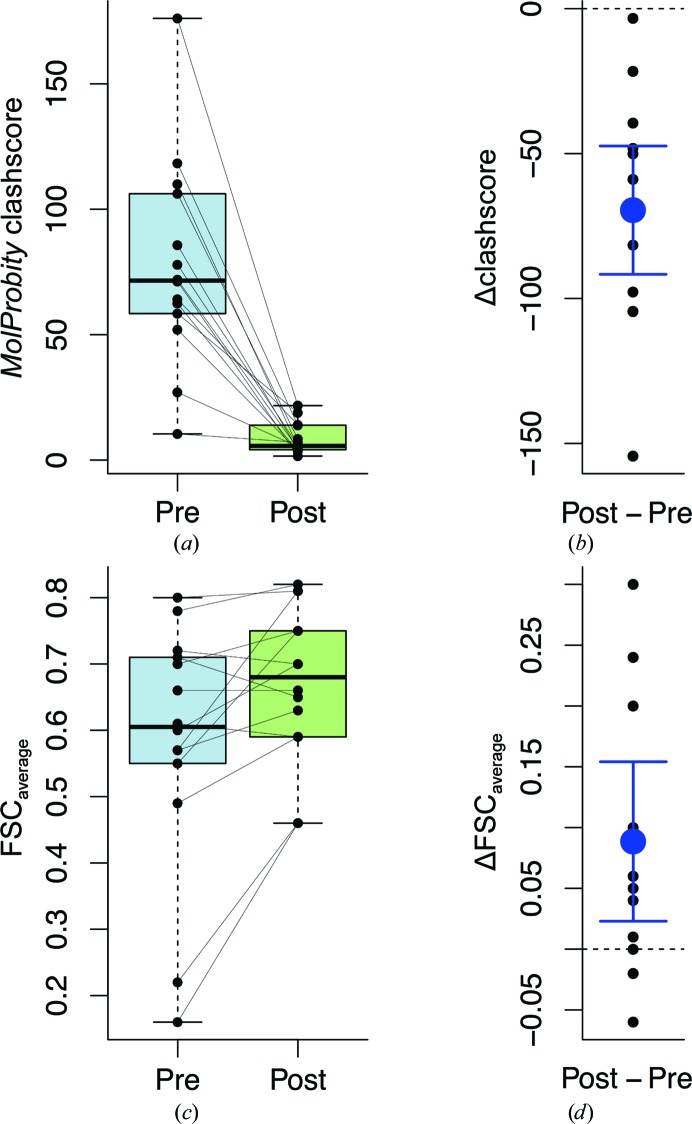
Box-and-whisker plots for refinement of EM structures at 4 Å resolution or better. (*a*) *MolProbity* clashscores before (pre) and after (post) restrained refinement with *REFMAC*. (*b*) Improvement in clashscores showing the mean of the differences and 95% confidence intervals. (*c*) FSC_average_ before and after restrained refinement. (*d*) Improvement in FSC_average_ showing the mean of the differences and 95% confidence intervals.

We have previously applied restrained refinement with *REFMAC* to ribosome structures solved by cryo-EM (Amunts *et al.*, 2014 ▶; Fernández *et al.*, 2014 ▶; Wong *et al.*, 2014 ▶). To demonstrate that this approach can be used on a diverse range of structures, EM maps with a reported resolution of 4 Å or better were obtained from the Electron Microscopy Data Bank (EMDB; release 2014-03-26; Lawson *et al.*, 2011 ▶) and the associated models from the PDB (Berman *et al.*, 2002 ▶). Maps not associated with a full-atom model were discarded, and an additional four maps were removed for technical reasons. Higher resolution structures that could act as reference models for restrained refinement were obtained using a search of the PDB for structural similarity (Krissinel & Henrick, 2004 ▶). Prior to refinement, each model was inspected for reasonable geometry, conformation and sterics using *MolProbity* (Chen *et al.*, 2010 ▶) and for fit to density using FSC_average_ (Fig. 9 ▶). Deposited models show a great variation in the *MolProbity* clashscore, which is the number of clashes per 1000 atoms, with clashes declared at an overlap of ≥0.4 Å. The clashscores are typically worse relative to structures solved by X-ray crystallography within a similar resolution cohort and lie at the 30th percentile. Only 20% of structures are annotated as having undergone any form of refinement. Each model was then subjected to two rounds of refinement in *REFMAC* with reference (when applicable) and secondary-structure restraints applied. Each round of refinement consisted of 20 cycles with external restraints regenerated between rounds. In cases where the models were of symmetric species, only the repeat unit was refined. Refinement improved the clashscore for all of the structures and improved the fit to density in all but three cases (Fig. 9 ▶). These cases were potentially overfitted prior to refinement, or the default refinement procedure was not adequate to improve the fit to density. The clashscore was lowered by a statistically significant average of 69.5 points (*p* = 6.5 × 10^−6^; paired *t*-test), with all models occupying a percentile better than 90 (with an average of 98.5). The fit to density, as measured by FSC_average_, improved from a mean of 0.58 to 0.67 (*p* = 6.0 × 10^−3^; paired *t*-test). Overfitting could not be examined as it is not yet common practice to deposit half maps.

As an example, we refined the structure of the heterotrimeric repeat unit of F420-reducing [NiFe] hydrogenase (Frh) from a hydrogenotrophic methanogenic archaeon (PDB entry 4ci0) against the deposited map at 3.34 Å resolution (EMD-2513; Allegretti *et al.*, 2014 ▶). Reference restraints were generated from other [NiFe] hydrogenases resolved at higher resolution and secondary-structure hydrogen-bond and helical fragment restraints were generated for the complete heterotrimer. The quality of the model was examined before and after refinement using *MolProbity* (Chen *et al.*, 2010 ▶). All statistics improved (Table 2 ▶), with the exception of the Ramachandran outliers, presumably as the dihedral angle restraints applied during model building can position backbones into incorrect local minima.

## 6. Validation   

Reference bias refers to a common problem in fitting experimental data to an initial model and is usually monitored using cross-validation, where the data used to assess the validity of the fit should not be the same as the data used to perform the fitting and should be independent of one another. In X-ray crystallography this is achieved by setting aside a random set of reflections (typically 5–10%; Brünger, 1992 ▶) that are preserved purely for cross-validation and are not used in refinement. If the model truly fits the data then the excluded reflections should also agree with the model. However, in cryo-EM structure factors can be strongly correlated and setting aside a random and independent selection is not achievable. A number of cross-validation methods analogous to those used in crystallography have been described for EM, including splitting the data into two independent data sets of which only one is used for model building and refinement (Shaikh *et al.*, 2003 ▶), exclusion of resolution shells in reciprocal space (DiMaio *et al.*, 2013 ▶) and omitting data from the high spatial frequency range (Falkner & Schröder, 2013 ▶). However, these approaches have yet to be widely adopted by the EM community, presumably as the more signal that is omitted during refinement the lower the quality of the refined structure.

**Figure 10:**
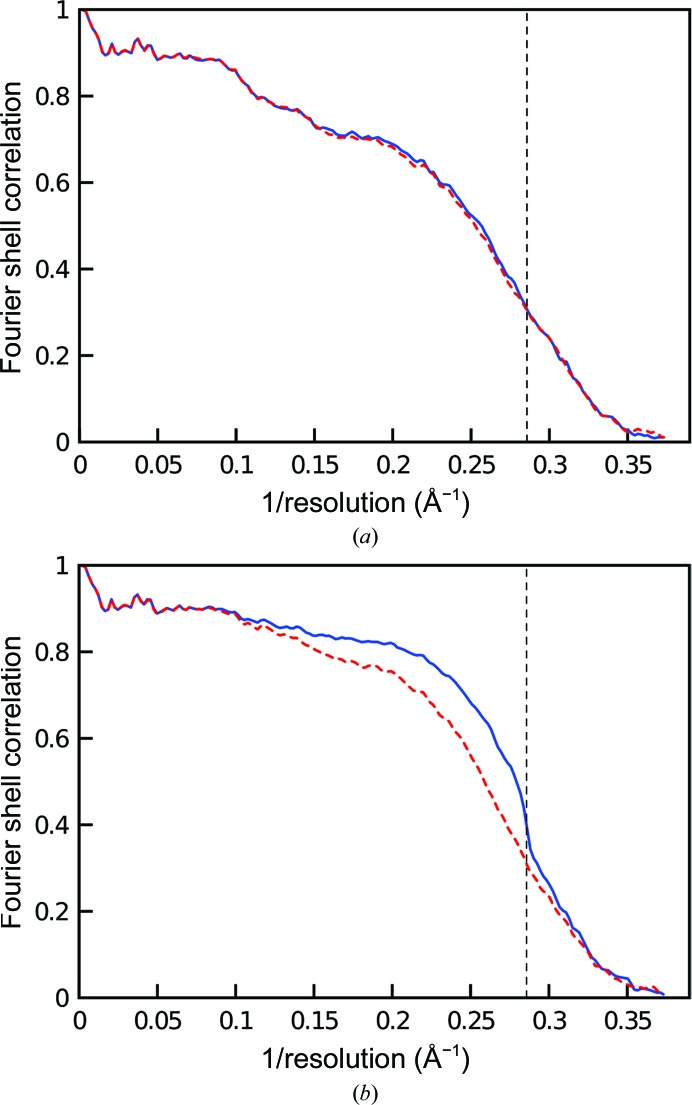
Effect of overfitting on FSC curves. (*a*) A refined structure that does not display the hallmarks of overfitting. FSC_work_ is shown with a continuous blue line and FSC_test_ with a dashed red line. The resolution cutoff applied during refinement is shown as a vertical dashed line. (*b*) An overfitted structure showing disagreement between FSC_work_ and FSC_test_ and a sharp decrease at the resolution limit applied during refinement.

We have previously described an approach to validate overfitting that does not require data to be omitted during the building/refinement process, but rather makes use of the two independent ‘half maps’ that are calculated from the same halves of the particles as used for the ‘gold-standard’ FSC calculations (Amunts *et al.*, 2014 ▶). This procedure involves an initial random displacement of atoms within a model to remove model bias before a fully restrained refinement against one of the two half maps. For each refinement, in addition to calculating the FSC between the refined model and the map that it was refined against (FSC_work_), a cross-validated FSC is calculated between the refined model and the other half map (FSC_test_). Large differences between FSC_work_ and FSC_test_ are indicative of overfitting. In addition, a sharp drop in FSC_work_ at the highest resolution that was included in the refinement also indicates overfitting, as it demonstrates a loss of the predictive power of the model. To illustrate the effect of overfitting on FSC curves, we added noise to the atoms of the final 54S model and re-refined with reduced geometric restraint weights and no external restraints against the 3.37 Å reconstruction (Fig. 10 ▶).

During post-processing the final reconstruction may undergo masking, modulation transfer-function correction of the imaging detector and *B*-factor sharpening to improve the appearance of the map. As a result, the half maps and the final summed map have different levels of sharpening that need to be put onto the same scale for cross-validation. We have therefore implemented into *REFMAC* automated reference-structure sharpening that enables maps to be placed on the same scale as either a reference curve or a reference map (*i.e.* the final reconstruction). Reference sharpening only works if one map is used for map calculation. By homogenizing maps, this should simplify the process of cross-validation and prevent inconsistencies.

## 7. Discussion   

Single-particle cryo-EM is a rapidly developing technique that is now capable of delivering structures at resolutions similar to those achieved by X-ray crystallography. However, software for interpreting these reconstructions with stereochemically reasonable atomic models has lagged behind. Here, we have presented a number of new tools to facilitate the interpretation of EM maps, from initial density-based fold identification through model building to refinement and validation. Many of these tools have been adapted from those used for X-ray crystallography and made suitable for EM maps, and are distributed through the *CCP*4 suite (Winn *et al.*, 2011 ▶). The CCP-EM project has been initiated to facilitate this cross-talk with CCP4 (Wood *et al.*, 2015 ▶).

Perhaps the greatest challenge in the interpretation of EM data is that of heterogeneity between multiple reconstructions that can be obtained from the same data set and variations in local resolution within each reconstruction. This means that global refinement strategies are not necessarily satisfactory and there is a potential need for ‘multi-resolution modelling’ that incorporates prior knowledge and complementary data from other experimental techniques (Villa & Lasker, 2014 ▶) and is applied at a local level. While we have implemented methods to optimize refinement protocols against segmented, composite and averaged maps and to apply weights to external restraints on the basis of local requirements, further exploration is required into localized tuning of external and/or geometry restraint weights based on local resolution (and other factors).

For optimizing the fit of models into EM maps, it is necessary to calculate the ‘observed’ variance of Fourier coefficients for use in refinement. This will reduce the fit of model parameters into noise and thus increase the reliability of derived atomic models. Another outstanding issue, the importance of which should not be underestimated, is that neither errors of density amplitudes on grid points in real space nor individual structure factors in reciprocal space are independent. This problem needs to be fully addressed; however, it seems that iterating between real-space and reciprocal-space refinement partially addresses it. As shown in Appendix *A* [App appa], weighted refinement in real space is equivalent to multivariate refinement in reciprocal space and *vice versa*. Thus, by selecting accurate weights (related to the inverse variances of EM maps) in real and reciprocal space this problem can partially be circumvented.

Proper validation of EM reconstructions and models built into EM maps is of increasing importance (Henderson *et al.*, 2012 ▶). For this purpose, we have described a method of validation that utilizes the two independent half maps produced during image processing. Alongside final reconstructions and structural models, the deposition of independent half maps and masks is strongly encouraged.

## Figures and Tables

**Table 1:** Particles used to generate 54S reconstructions at different resolutions

Particles	Resolution ()
1000	6.79
1500	5.68
2000	5.03
3000	4.43
5000	4.05
7000	3.89
10000	3.71
20000	3.50
48000	3.37

**Table 2:** Refinement statistics for the Frh heterotrimer Reference restraints were generated for the FrhA and FrhG subunits using PDB entries 3ze6 and 3uqy, respectively. Secondary-structure hydrogen-bond and helical fragment restraints were generated for the complete heterotrimer. After 20 cycles of restrained refinement, the restraints were regenerated and applied for a second set of 20 cycles.

	Pre-refinement	Post-refinement
FSC_average_	0.78	0.82
*R* factor (weighted, overall)	0.34	0.31
Average *B* factor (^2^)	n.d.	96.5
R.m.s.d., bonds ()	0.017	0.007
R.m.s.d., angles ()	2.40	1.91
*MolProbity* score (percentile)	3.9 (30th)	2.91 (91st)
Clashscore, all atoms (percentile)	118.3 (5th)	13.9 (97th)
Good rotamers (%)	64.5	88.3
Ramachandran outliers (%)	0.11	1.35

## Appendix A On the similarity of real-space and reciprocal-space refinement

In this appendix, we demonstrate that, in essence, refinement in real space and reciprocal space are similar. We use the following notation: bold letters are vectors, **h** is a reciprocal-space vector, **x** is a real-space vector, **F** is a complex Fourier coefficient, *s* is the length of the reciprocal-space vector in an orthogonal coordinate frame corresponding to the index **h**,  denotes the Fourier transformation and  denotes the inverse Fourier transformation. To simplify the equations, we assume that ρ_1_(**x**) and its reciprocal-space counterpart **F**_1**h**_ correspond to the observed map and structure factors, ρ_2_(**x**) and its reciprocal-space counterpart **F**_2**h**_ are the map and structure factors corresponding to an atomic model, and all definable parameters including an overall scale are included in ρ_2_(**x**) or **F**_2**h**_.

Let us assume that we have two maps, ρ_1_(**x**) and ρ_2_(**x**), in a box with volume *V*. Let us denote their Fourier transformations **F**_1**h**_ =  and **F**_2**h**_ = . According to Parseval’s theorem (Rudin, 1991 ▶),

and for the discrete version of this relationship (in practice we work with discretized versions of maps, so the following relationship is more relevant),

where *N*_1_, *N*_2_ and *N*_3_ are the number of grid points in three orthogonal directions.

The left-side summation is over grid points in real space and the right-side summation is over all reciprocal-space vectors **h** within the resolution range. Note that the limits of **h** are defined by the resolution of the map, whereas in real space the grid sampling can be as fine as desired. Consequently, we can assert that unweighted least-squares minimization in real space is equivalent to least-squares minimization in reciprocal space. One of the advantages of using minimization in reciprocal space is that it is relatively straightforward to design weights,

where *w*_**h**_ = 1/Σ(**h**) is a weight, Σ(**h**) = 〈|**F**_1**h**_ − **F**_2**h**_|^2^〉 is the variance of differences between structure factors and 〈.〉 is the expectation operator.

This formulation is essentially equivalent to using the log-likelihood function based on the conditional distribution of observed complex structure factors given calculated structure factors (Luzzati, 1953 ▶). Note that weighted least squares in reciprocal space is not directly related to weighted least squares in real space. If we use Parseval’s theorem followed by the convolution theorem, we can see that

where *W*(**x**) =  is the inverse Fourier transformation of the weights used in reciprocal space and * denotes complex conjugation.

In the summation, both **x** and **y** run over all grid points in the box. This relationship shows that using weighted least squares in reciprocal space is equivalent to using multivariate least squares in real space, *i.e.* accounting for the correlation between all points in the map. Since Parseval’s theorem and the convolution theorem work for forward as well as backward Fourier transformations, it can be seen that using weighted least squares in real space is equivalent to using multivariate least squares in reciprocal space. It seems that although reciprocal-space and real-space refinements are similar, it might sometimes be more convenient to design weights in one space or the other. Iterating between real-space and reciprocal-space weighted least-squares fitting might allow one to derive an optimal model that explains the experimental data.

## Appendix B On the dependence of the overall *R* factor and the Fourier shell correlation on refinement weights

Notation used in this appendix: **h** is the reciprocal-space vector with length *s*, **S** is a reciprocal-space sphere with radius *s* and d**S** is an element of this sphere.

It is common practice to control refinement behaviour using either correlation or *R* factors. In this appendix, we demonstrate that one should be careful in using such overall indicators. When weights in refinement change, these indicators are no longer comparable. Such weights can either be by design or implicit. For example, using different sharpening during refinement is equivalent to multiplication of the structure factors **F_h_** by exp(−*Bs* ^2^/4). Therefore, calculating overall correlation and *R* factors is equivalent to using weighted correlation or *R* factors. The overall weighted *R* factor is given by

and the overall weighted FSC is given by

When no weights are used then *w*_**h**_ = 1.

Note that when calculated using different weights these statistics are not equivalent. An extreme case is when the weight corresponding to one reflection (**k**) is 1 and all others are 0,

In this case the FSC will be cos(ϕ_1**k**_ − ϕ_2**k**_) and the *R* factor will depend on only one reflection.

It is easy to see that when using different overall *B* factors in refinement (map sharpening or blurring) we are essentially using weights for the calculation of the overall *R* factor and FSC: for the overall *R* factor we use *w*_**h**_ = exp(−*Bs* ^2^/4) and for the overall FSC we use *w*_**h**_ = exp(−*Bs* ^2^/2). To avoid this dependence on weight, we prefer to use the average FSC,

where *N*_shell_ is the number of resolution shells used to calculate the FSC, FSC_*i*_ corresponds to the *i*th shell and *N*_*i*_ is the number of structure factors in the *i*th shell. If the resolution shells are sufficiently thin then the weights of all structure factors within each shell will be roughly equal to each other. Since the same weights are on the denominator and numerator of the expressions for the *R* factor and correlation, they will cancel out, and FSC_average_ will be independent of weight. In the limiting case when a shell width goes to 0, and if we assume that the reciprocal-space points are sufficiently dense, then FSC_average_ would converge to the following integral:

where FSC(*s*) is calculated on the surface of a reciprocal-space sphere of radius *s*,

where *s*_min_ and *s*_max_ are the resolution limits used in FSC calculations, integration is over the reciprocal-space surface of the sphere **S** of radius *s* and d**S** represents a surface element.

If the weights are dependent only on the length of the reciprocal-space vector (as is the case for effective weights owing to the overall *B* factor) then it is seen that each FSC(*s*) is independent of the weight, and therefore the average FSC is also independent of such weights.

## Appendix C Parallel-plane restraints

Notation used in this appendix: bold letters are three-dimensional vectors, **uv** ^T^ = *u*_1_ *v*_1_ + *u*_2_ *v*_2_ + *u*_3_ *v*_3_ is the scalar product of two three-dimensional vectors, |**u**| = (**uu** ^T^)^1/2^ is the length of the three-dimensional vector, (**a**, *d*) defines a plane and the equation of a plane is **ax** ^T^ − *d* = 0 for all *x* ∈ *R* ^3^.

Let us assume that we have two sets of atoms {**x**_11_, **x**_12_ …, **x**_1*n*_} and {**x**_21_, **x**_22_ …, **x**_2*m*_}. We want each set to be on a plane and these planes to be parallel. Mathematically, this can be expressed in various ways, two of which are the following.

### c1. Pooled-atoms plane

We would like two planes to be parallel. This is equivalent to the minimization

In this case, by construction, the coefficients of the planes for both sets of atoms will be the same. Consequently, parallelity of the resultant planes is guaranteed. This formulation has several attractive features: (i) it is easy to implement, (ii) the number of planes is not limited and (iii) if conjugate-gradient or similar minimization is used then it is not necessary to use derivatives of eigenvalues and eigenvectors with respect to *x*_*j*,*i*_.

### c2. Angles between planes

In this case, restraints are expressed as (we assume that the angle between planes should be α_0_)

where α is the current angle between planes formed by (**a**_1_, *d*_1_) and (**a**_2_, *d*_2_),

Note that if the lengths of the vectors **a**_*i*_ are equal to 1, *i.e.* |**a**_*i*_| = 1, then this expression has an especially simple form.

The first step of implementing parallel-plane restraints involves solving the following minimization problem,

with respect to (**a**_*j*_, *d*_*j*_), under the condition that |**a**_*j*_| = 1. Note that the resulting (**a**_*j*_, *d*_*j*_) are dependent on **x**_*j*,*i*_. This problem is solved by finding eigenvalues and eigenvectors of the matrix

where **X**_*j*_ is a matrix built by using vectors **x**_*j*,*i*_ −  row-wise and  is the weighted average (or centre of mass) over all **x**_*j*,*i*_.

Eigenvectors corresponding to the smallest eigenvalue of this matrix are those corresponding to **a**_*j*_. Once **a**_*j*_ are known then *d*_*j*_ is calculated in a straightforward manner,

By construction, |**a**_*j*_| = 1.

It can be seen that **a**_*j*_ and *d*_*j*_ are dependent on **x**_*j*,*i*_. In general, for planarity restraints these dependencies need to be accounted for. If only the conjugate-gradient or a similar first-order minimization method is used then it can be shown that the dependence of **a**_*j*_ and *d*_*j*_ on **x**_*j*,*i*_ can be ignored. However, this is not the case if second-order minimization methods are used. In order to account for these dependences, it is necessary to use derivatives of **a**_*j*_ and *d*_*j*_ with respect to **x**_*j*,*i*_. These derivatives are calculated using the method described in Nelson (1976 ▶).

Once the derivatives of **a**_*j*_ with respect to **x**_*j*,*i*_ are available, we can calculate the derivatives of α − α_0_ with respect to the atomic parameters using the chain rule.

This formulation has the attractive feature that we can restrain the angles between two planes to any desired angle. For example, if we know that π stacking between two planes is T-shaped then we can set α_0_ = 90°. As a rule, RNA/DNA bases form parallel stacking and thus α_0_ = 0 must be set.

### c3. Parallel-plane restraints in *Coot*

The handling of parellel-plane restraints in *Coot* is rather more simplistic. The planar system restraint (18) was extended to permit parallel-plane restraints. The simple plane-restraint system minimizes *S*_plane_,

where *e_ij_* is the deviation of the *j*th atom in the *i*th plane from the plane restraint’s least-squares plane.

This was extended so that the pairs of planes could be restrained to be parallel. The set of atoms comprising each of the plane systems of a parallel-plane pair is moved to the origin and there a new pseudo-plane system is generated comprising the set of atoms of each plane system. The planar distortion and plane gradients of each atom from this pseudo-plane are calculated,

where *e_ij_* and *e_ik_* are the deviations of the atoms in the *i*th pseudo-plane from the pseudo-plane restraint’s origin-centred least-squares plane. *N*_p1_ and *N*_p2_ are the number of atoms in each of the the individual planes contributing to a parallel-plane pair.
